# Supplementary material for: Mapping the evolution of fertility support policies in China: A content and instrumental analysis
Source: PLoS One. 2025 Oct 9;20(10):e0332137. doi: 10.1371/journal.pone.0332137 (PMC12510515; doi:10.1371/journal.pone.0332137)
Supplement: S1 Appendix — (ZIP) [file pone.0332137.s001.zip › S1 Appendix. 226 original policy documents/118-商务部办公厅关于促进社区消费 切实解决老年人运用智能技术困难的通知(FBM-CLI-4-349532).doc]

商务部办公厅关于促进社区消费 切实解决老年人运用智能技术困难的通知

**发布部门：** [商务部](https://www.pkulaw.com/law/chl?Aggs.IssueDepartment=60220) [机构沿革](javascript:void(0))

**发文字号：**商办服贸函〔2020〕431号

**发布日期：**2020.12.28

**实施日期：**2020.12.28

**时效性：** [现行有效](https://www.pkulaw.com/law/chl?Aggs.TimelinessDic=01)

**效力级别：** [部门工作文件](https://www.pkulaw.com/law/chl?Aggs.EffectivenessDic=XE0303)

**法规类别：** [机关工作综合规定](https://www.pkulaw.com/law/chl?Aggs.Category=00301)

**专题分类：** [人工智能](https://www.pkulaw.com/law/chl?Aggs.SpecialType=007)

商务部办公厅关于促进社区消费 切实解决老年人运用智能技术困难的通知
（商办服贸函〔2020〕431号）

各省、自治区、直辖市、计划单列市及新疆生产建设兵团商务主管部门：
　　为深入贯彻习近平总书记关于切实解决老年人运用智能技术困难的重要批示精神，落实好《[国务院办公厅印发关于切实解决老年人运用智能技术困难实施方案的通知](https://www.pkulaw.com/chl/c29665300feed18bbdfb.html)》（国办发〔2020〕45号），促进社区便民消费，保障社区居家老年人生活需求，切实解决老年人运用智能技术的困难，现就有关工作通知如下：
　　**一、**总体要求
　　以习近平新时代中国特色社会主义思想为指导，全面贯彻党的十九大和十九届二中、三中、四中、五中全会精神，认真落实党中央、国务院决策部署，坚持以人民为中心的发展思想，坚定不移贯彻新发展理念，加快新建改造一批充分兼顾老年人需要的社区便民消费服务设施，按照传统服务方式与智能化服务创新“两条腿”走路的方式，保障老年人生活需求，提升老年人获得感、幸福感、安全感。

　　**二、**主要任务
　　（一）加快新建和改造一批城乡便民消费服务中心。各地要参照新修订的《社区便民服务中心建设指南》《街区生活服务集聚中心建设指南》《农村生活综合服务中心建设指南》，加快建设一批集餐饮、家政、托幼、老人看护等服务于一体的城乡便民消费服务中心，对现有城乡便民消费服务中心进行适老化改造，保留现金支付方式和面对面人工服务，优化智能服务方式，营造老年人友好型消费环境。
　　（二）加快发展社区品牌连锁便利店。支持便利店优化布局，加大门店建设拓展力度，推动便利店进街区社区。鼓励便利店在优化智能服务方式的同时，保留传统现金支付方式，搭载书报经营、代扣代缴、代收代发、家政预约、送货到家等服务项目，提升便民服务的“温度”。
　　（三）便利老年人日常消费。配合相关部门督导零售、餐饮、商场等场所要保留面对面人工服务，支持现金和银行卡支付。另一方面，引导上述场所提供简便易行、符合老年人特点的智能服务方式，便利老年人消费。

　　**三、**保障措施
　　（一）提高思想认识。解决老年人运用智能技术困难问题是践行“两个维护”的必然要求，是坚持以人民为中心的发展思想的重要体现，是落实国家积极应对人口老龄化中长期规划的重要举措。各地商务主管部门要高度重视老年人相关工作，增强使命感、责任感和紧迫感，加强横向协作和纵向联动，强化政策支持，出实招、办实事，明确责任人，制定时间表和路线图，确保各项工作取得实效。
　　（二）加强监督检查。各地商务主管部门要加强监督检查，指导已建城乡便民消费服务中心运营单位提升为老服务水平，配合有关部门督导零售、餐饮、商场等场所保留面对面服务和传统支付方式，查找突出问题和薄弱环节，不断完善工作举措。
　　（三）及时总结推广。各地要充分发挥主动性和创造性，及时总结可复制、可推广的经验和做法。各省级商务主管部门有关工作开展情况、建设成效、存在的问题和困难以及经验做法请于2021年6月30日前报送商务部（服贸司）。
　　联系电话： 010-85093778/3783
　　附件：1.《社区便民服务中心建设指南》
　　2.《街区生活服务集聚中心建设指南》
　　3．《农村生活综合服务中心建设指南》

商务部办公厅
2020年12月28日

　　附件1
社区便民服务中心建设指南

　　**一、**总体要求
　　（一）本建设指南所指社区便民服务中心是指以社区居民为主要服务对象，以满足社区居民基本生活消费和便民、利民需求为目标，提供公益性和商业性相结合的餐饮、家政、托幼、老人看护、维修等居民生活服务的便民服务集聚综合体。
　　本建设指南适用于城市社区便民服务中心的新建、改造及经营管理。
　　（二）社区便民服务中心必须坚决贯彻落实党中央、国务院有关要求，坚持以人民为中心的发展思想，符合国家相关法规标准，综合考虑经济社会发展水平，坚持传统服务方式与智能化服务创新“两条腿”走路，从居民特别是老年人、儿童、残疾人等群体日常生活需求出发，充分利用现有公共服务和商业设施，合理确定建设水平，做到服务便利、规模适宜、功能完善、安全卫生、运行经济。

　　**二、**建设规模及服务功能要求
　　（一）社区便民服务中心规模应以社区居住人口数量为主要依据，兼顾服务半径确定，营业面积300平方米以上。
　　（二）社区便民服务中心服务功能应与社区的人口结构、居民消费水平、习惯和方式相适应，以满足广大居民日常生活需求、便捷消费为目标，进一步增强对老年人、儿童、残疾人等群体生活服务的保障，具体分为基本服务和选择性服务，详见下表。新建社区便民服务中心应优先考虑配置基本服务功能，改造的社区便民服务中心应通过多种途径逐步完善基本服务功能。针对我国老龄化程度不断加深的趋势，社区便民服务中心应根据社区居民需求积极进行适老化改造，提高为老服务水平。
　　社区便民服务中心服务功能组合表

| 基本服务 | 选择性服务 |
| --- | --- |
| 便利店、生鲜菜店、大众餐饮（早餐）、家政、理发、维修 | 托幼、老人看护、洗衣、代收代缴、补衣缝纫、康体健身、美容美甲、活动室等 |

　　**三、**选址布局要求
　　（一）社区便民服务中心应选择服务对象相对集中，交通便利，供电、给排水、通讯等市政条件较好，符合消防、食品卫生等安全要求的场所。
　　（二）社区便民服务中心应根据各服务功能的特点，进行合理布局。

　　**四、**经营管理要求
　　（一）社区便民服务中心一般采用统一经营管理的方式，即各项服务的经营权和管理权完全纳入到一个组织机构下，统一规划设计、统一招商经营、统一运营管理，从源头上把控便民服务中心的功能组合、品牌档次，保障项目完整性，最大程度满足社区居民的服务需求。
　　（二）经营场所进行简洁装修，墙壁和地面便于经常清扫刷洗，店内通风、明亮。使用检定合格、未超过检定周期的计量器具。配备消防安全设施或设备，保证消防安全设施齐全、完好、有效。
　　（三）各岗位的从业人员应无传染性疾病，经营食品、副食品的从业人员还应定期进行健康检查，并持有当地卫生主管部门颁发的健康证明。各岗位的从业人员应具有一定的文化程度和与其岗位相适应的业务知识、技术水平，经过社会专门培训机构或企业组织的岗前培训并考试合格。
　　（四）制定职业道德守则，所有岗位从业人员都应遵守，社区便民服务中心定期对从业人员遵守职业道德守则情况进行认真考核。
　　（五）坚持传统服务方式与智能化服务创新“两条腿”走路，在积极通过信息化提升服务便利化水平的同时，要充分兼顾老年人需要，保留必要的面对面服务，保留现金和银行卡等传统支付方式，便利老年人社区消费，保障老年人生活消费需要。
　　（六）执行国家物价管理政策，所有商品和服务应明码标价。

　　附件2
街区生活服务集聚中心建设指南

　　**一、**总体要求
　　（一）本建设指南所指街区生活服务集聚中心是指为满足居民购物、美食、休闲娱乐等生活服务需求，实行统一管理并具有一定规模的生活服务集聚街区，按主要服务功能可分为购物街、美食街、休闲娱乐街和综合生活服务街。
　　本建设指南适用于中心城市街区生活服务集聚中心的新建、改造和经营管理。
　　（二）街区生活服务集聚中心必须坚决贯彻落实党中央、国务院有关要求，坚持以人民为中心的发展思想，符合国家相关法规标准，综合考虑经济社会发展水平，坚持传统服务方式与智能化服务创新“两条腿”走路，从城市居民日常生活需求出发，充分利用现有公共服务和商业设施，合理确定建设水平，做到服务便利、规模适宜、功能完善、安全卫生、运行经济。

　　**二、**建设规模及服务功能要求
　　（一）街区的总长度一般应在400米以上，宽度可按街区内设施和人流量确定。
　　（二）街区主要服务功能可以是购物、美食、休闲娱乐等生活服务功能中的一种，也可以是几项生活服务功能的组合。

　　**三、**选址布局要求
　　（一）街区生活服务集聚中心应选择服务对象相对集中，交通便利，供电、给排水、通讯等市政条件较好，符合消防、食品卫生等安全要求的场所。
　　（二）符合所在城市总体规划和商业网点规划的要求，避免盲目冒进、重复建设。

　　**四、**经营管理要求
　　（一）经营场所进行简洁装修，墙壁和地面便于经常清扫刷洗，店内通风、明亮。使用检定合格、未超过检定周期的计量器具。配备消防安全设施或设备，保证消防安全设施齐全、完好、有效。
　　（二）各岗位的从业人员应无传染性疾病，经营食品、副食品的从业人员还应定期进行健康检查，并持有当地卫生主管部门颁发的健康证明。各岗位的从业人员应具有一定的文化程度和与其岗位相适应的业务知识、技术水平，经过社会专门培训机构或企业组织的岗前培训并考试合格。
　　（三）制定职业道德守则，所有岗位从业人员都应遵守，街区生活服务集聚中心定期对从业人员遵守职业道德守则情况进行认真考核。
　　（四）执行国家物价管理政策，所有商品和服务应明码标价。
　　（五）坚持传统服务方式与智能化服务创新“两条腿”走路，在积极通过信息化提升服务便利化水平的同时，要充分兼顾老年人需要，保留必要的面对面服务，保留现金和银行卡等传统支付方式，便利老年人消费。不得以格式条款、通知、声明、告示等任何方式拒收现金。

　　附件3
农村生活综合服务中心建设指南

　　**一、**总体要求
　　（一）本建设指南所指农村生活综合服务中心是指以农村乡镇、自然村或行政村居民为主要服务对象，以满足居民基本生活消费和便民、利民需求为目标，提供公益性和商业性相结合的大众化居民生活服务的便民服务集聚综合体，按所在位置、规模和服务对象可分为乡（镇）生活综合服务中心和村级生活综合服务站。
　　本建设指南适用于农村生活综合服务中心的新建、改造和经营管理。
　　（二）农村生活综合服务中心项目必须坚决贯彻落实党中央、国务院有关要求，坚持以人民为中心的发展思想，符合国家相关法规标准，综合考虑农村区域经济社会发展水平，从农村居民特别是老人、儿童、残疾人等群体日常生活需求出发，充分利用现有公共服务和商业设施，合理确定建设水平，做到服务便利、规模适宜、功能完善、安全卫生、运行经济。

　　**二、**建设规模及服务功能要求
　　（一）农村生活综合服务中心项目规模应以乡镇、自然村或行政村居住人口数量为主要依据，兼顾服务半径确定。乡（镇）生活综合服务中心营业面积要求：东中部地区300平方米以上，西部地区200平方米以上。村级生活综合服务站营业面积要求：东中部地区100平方米以上，西部地区80平方米以上。
　　（二）农村生活综合服务中心服务功能应与乡镇、自然村或行政村的人口结构、居民消费水平、习惯和方式相适应，应以满足居民日常生活需求、便捷消费为目标，进一步增强对老年人、儿童、残疾人等群体生活服务的保障，具体分为基本服务和选择性服务，详见下表。新建农村生活综合服务中心应优先考虑配置基本服务功能，改造的农村生活综合服务中心应通过多种途径逐步完善基本服务功能。
　　农村生活综合服务中心服务功能组合表

| 乡（镇）生活综合服务中心 | 基本服务 | 选择性服务 |
| --- | --- | --- |
|  | 大众餐饮（早餐）、维修、理发、生鲜菜店、便利店 | 家政、托幼、老人看护、洗衣、代收代缴、补衣缝纫、美容美甲、活动室等 |
| 村级生活  综合服务站 | 基本服务 | 选择性服务 |
|  | 便利店、维修、理发 | 大众餐饮（早餐）、家政、托幼、老人看护、洗衣、代收代缴、补衣缝纫、美容美甲、活动室等 |

　　**三、**选址布局要求
　　（一）农村生活综合服务中心应选择服务对象相对集中，交通便利，供电、给排水、通讯等市政条件较好，符合消防、食品卫生等安全要求的场所。
　　（二）农村生活综合服务中心应根据服务功能的特点，进行合理布局。

　　**四、**经营管理要求
　　（一）农村生活综合服务中心一般采用统一经营管理的方式，即各项服务的经营权和管理权完全纳入到一个组织机构下，统一规划设计、统一招商经营、统一运营管理，从源头上把控农村生活综合服务中心的功能组合、品牌档次，保障项目完整性，最大程度满足居民的消费服务需求。
　　（二）经营场所进行简洁装修，墙壁和地面便于经常清扫刷洗，店内通风、明亮。使用检定合格、未超过检定周期的计量器具。配备消防安全设施或设备，保证消防安全设施齐全、完好、有效。
　　（三）各岗位的从业人员应无传染性疾病，具有一定的文化程度，经过岗前培训。
　　（四）执行国家物价管理政策，所有商品和服务应明码标价。
　　（五）坚持传统服务方式与智能化服务创新“两条腿”走路，充分兼顾农村老年人需求，保留必要的面对面服务，保留现金和银行卡等传统支付方式，保障老年人生活消费需要。

© 北大法宝：（[www.pkulaw.com](https://www.pkulaw.com/)) 专业提供法律信息、法学知识和法律软件领域各类解决方案。北大法宝为您提供丰富的参考资料，正式引用法规条文时请与标准文本核对。
欢迎查看所有[产品和服务](https://www.pkulaw.com/helps/2.html)。法宝快讯：[如何快速找到您需要的检索结果？](https://www.pkulaw.com/helps/70.html)[法宝](https://www.pkulaw.com/helps/69.html)V6有何新特色？

扫描二维码阅读原文

**原文链接：**<https://www.pkulaw.com/chl/5d388f12bc42251bbdfb.html>
